# Peer Review of “Challenges in Implementing a Mobile AI Chatbot Intervention for Depression Among Youth on Psychiatric Waiting Lists: Randomized Controlled Study Termination Report”

**DOI:** 10.2196/82073

**Published:** 2025-09-05

**Authors:** Maria da Graca Ambrosio

**Affiliations:** 1 University of Oxford, Oxford, United Kingdom

**Keywords:** randomized controlled trial, AI chatbot, acceptance and commitment therapy, mental health, psychiatry, children, adolescents, Japan

*This is a peer-review report for “Challenges in Implementing a Mobile AI Chatbot Intervention for Depression Among Youth on Psychiatric Waiting Lists: Randomized Controlled Study Termination Report.”*

## Round 1 Review

### General Comments

This paper [1] describes the results of a parallel group randomized controlled trial that examined the feasibility of an artificial intelligence (AI) chatbot-led mental health intervention to support pediatric patients on the psychiatry waitlists in Japan. The article is well-written and organized, and the objectives of the study are clearly stated. Methodology elements such as eligibility criteria, information sources, and data collection process are clear. A clear list of outcomes and variables for which data were researched is presented. The authors provide an important contribution to the field by reporting on factors that challenge adolescents’ engagement in digital mental health interventions and providing meaningful recommendations for future research.

### Specific Comments

#### Major Comments

1. How many chatbots were shortlisted, and why was emol favored over the others, given the selection criteria? (Under AI Chatbot Selection Process.)

2. How are the six core processes of acceptance and commitment therapy delivered in the AI chatbot (under Intervention Group)? Expand more on each section. How does the session meet the core processes of acceptance and commitment therapy—acceptance, cognitive defusion, being present, self as context, values, and committed action?

3. How was the section structured? Did adolescents go through modules? Could they write anything to the chatbot, or was the content predefined? Were the sessions sequentially delivered or not? Could they access previously completed modules or track their progress?

4. Were there any safeguarding links and referral contacts built into the chatbot in case participants needed additional support beyond those offered by the chatbot? If yes, I recommend including it under the ethics paragraph.

5. How were you planning to investigate engagement? Would you report on the frequency of use, number of interactions with the chatbot, or amount of content visualized by participants? Even though the study’s main questions are not focused on engagement, I suggest that the authors consider including an engagement outcome paragraph right after the secondary outcomes.

#### Minor Comments

6. I recommend moving all hyperlinks to the appendix and including an image of the chatbot. I also recommend that authors include an image of the intervention delivered through the hospital website.

7. Please state the statistical methods used to deal with missing data.

8. In the Discussion, you argue that young people prefer online mental health support over in-person support [2]. I believe you could discuss this a bit more in your Introduction paragraph to strengthen your discussion regarding the potential gap online services could fill.

9. I recommend including a paragraph under the Introduction on previous Japanese studies focusing on chatbot-led or digital mental/public health interventions to provide an overview of the current population uptake of digital health interventions.

## Abbreviations

AI: artificial intelligence
